# β-Adrenergic Receptor Desensitization/Down-Regulation in Heart Failure: A Friend or Foe?

**DOI:** 10.3389/fcvm.2022.925692

**Published:** 2022-07-01

**Authors:** Abrahim Mahmood, Kinza Ahmed, Youhua Zhang

**Affiliations:** Department of Biomedical Sciences, New York Institute of Technology College of Osteopathic Medicine, Old Westbury, NY, United States

**Keywords:** β-adrenergic receptor, β-adrenergic receptor desensitization/down-regulation, cardiac ryanodine receptor, calcium leak, arrhythmia, heart failure

## Abstract

Cardiac sympathetic activation, mediated by β-adrenergic receptors (β-ARs), normally increases cardiac contraction and relaxation. Accomplishing this task requires a physiological, concerted Ca^2+^ signaling, being able to increase Ca^2+^ release from sarcoplasmic reticulum (SR) in systole and speed up Ca^2+^ re-uptake in diastole. In heart failure (HF) myocardial β-ARs undergo desensitization/down-regulation due to sustained sympathetic adrenergic activation. β-AR desensitization/down-regulation diminishes adrenergic signaling and cardiac contractile reserve, and is conventionally considered to be detrimental in HF progression. Abnormal Ca^2+^ handling, manifested as cardiac ryanodine receptor (RyR2) dysfunction and diastolic Ca^2+^ leak (due to sustained adrenergic activation) also occur in HF. RyR2 dysfunction and Ca^2+^ leak deplete SR Ca^2+^ store, diminish Ca^2+^ release in systole and elevate Ca^2+^ levels in diastole, impairing both systolic and diastolic ventricular function. Moreover, elevated Ca^2+^ levels in diastole promote triggered activity and arrhythmogenesis. In the presence of RyR2 dysfunction and Ca^2+^ leak, further activation of the β-AR signaling in HF would worsen the existing abnormal Ca^2+^ handling, exacerbating not only cardiac dysfunction, but also ventricular arrhythmogenesis and sudden cardiac death. Thus, we conclude that β-AR desensitization/down-regulation may be a self-preserving, adaptive process (acting like an intrinsic β-AR blocker) protecting the failing heart from developing lethal ventricular arrhythmias under conditions of elevated sympathetic drive and catecholamine levels in HF, rather than a conventionally considered detrimental process. This also implies that medications simply enhancing β-AR signaling (like β-AR agonists) may not be so beneficial unless they can also correct dysfunctional Ca^2+^ handling in HF.

## Introduction

Beta-adrenergic receptors (β-ARs) are members of a large family of cell surface receptors known as G-protein-coupled receptors (GPCRs) (1). β-ARs mediate the sympathetic adrenergic effect (the fight or flight response) on the heart. There are 3 β-AR subtypes: β1-AR, β2-AR and β3-AR in the heart (2). These receptors are predominantly found in the myocardium, vascular smooth muscle, and adipose tissue, respectively. Cardiomyocytes express all 3 subtypes, with β1-ARs being the predominant subtype, representing ~80%, whereas β2-ARs and β3-ARs comprising the remaining 17 and 3%, respectively (3, 4). In cardiomyocytes, β1-ARs are widely distributed across the cell membrane and can be activated by both norepinephrine (released from sympathetic nerve fibers) and epinephrine (from the chromaffin cells of the adrenal medulla) (5). Activation of β1-ARs and the subsequent Gs-protein coupled signaling cascade (adenylyl cyclase-cAMP-protein kinase A) leads to increased myocardial contraction (inotropy), relaxation (lusitropy), heart rate (chronotropy) and AV conduction (dromotropy), respectively (6). β2-ARs are more localized within the transverse (T)-tubules (7), have a higher affinity for epinephrine and are coupled to the Gs and Gi subunits, allowing for both stimulatory and inhibitory effects on cardiac contractile function (8). β3-ARs are expressed at low levels on cardiomyocytes, and their physiological role is less clear (9).

Heart failure (HF) is a global epidemic problem, affecting more than 37.7 million individuals globally and imposes substantial economic burden to the health care system (10). HF occurs when various assaults result in myocardial injury/loss, impairing cardiac function. In response, the neurohumoral system, particularly the sympathetic nervous system is activated. The sympathetic activation, mediated by the β-AR (mainly β1-AR) signaling cascade (as mentioned before), increases cardiac contractility, relaxation, and cardiac output. Thus, sympathetic activation acutely (or in short term) is a beneficial compensatory process. However, sustained long-term sympathetic activation in HF can lead to pathological remodeling in β-ARs, manifested as uncoupling of the β-AR signaling (desensitization) and reduced number of β-ARs (mainly β1-ARs) on cell membrane (β-AR down-regulation) (6). β-AR desensitization/down-regulation diminishes adrenergic signaling and contractile reserve, and is conventionally considered to be detrimental in HF deterioration/progression (3, 11, 12). This notion is further supported by the fact that effective treatments (e.g., beta-blockers) are associated with re-sensitization/up-regulation of the β-ARs (6). As a result, re-sensitizing/up-regulating β-ARs is currently believed to be beneficial and efforts are directed to develop medications to up-regulate/re-sensitize the β-ARs as therapeutic strategies in HF (6, 12, 13).

Abnormal Ca^2+^ signaling has been identified as another form of pathological remodeling in HF (14). Several critical Ca^2+^ handling components are dysfunctional in HF, including sarcoplasmic reticulum (SR) cardiac ryanodine receptor (RyR2, the calcium release channel) dysfunction and diastolic Ca^2+^ leak, reduced Ca^2+^ pump (the sarcoplasmic/endoplasmic reticulum Ca^2+^ ATPase, SERCA) activity, etc. (14). RyR2 dysfunction and Ca^2+^ leak could lead to depleted SR Ca^2+^ stores and elevated cytoplasmic Ca^2+^ levels in diastole, resulting in both systolic and diastolic dysfunction. In addition, by activating the inward Na^+^/Ca^2+^ exchanger (NCX) current (15), diastolic Ca^2+^ leak and elevated cytoplasmic Ca^2+^ levels could depolarize membrane potential, contributing to electrical instability, delayed afterdepolarizations, triggered arrhythmias, and sudden cardiac death (16–19). RyR2 dysfunction and Ca^2+^ leak in HF is caused by sustained sympathetic activation and elevated β-AR signaling (14). Thus, it is foreseeable that further activation of β-AR signaling would worsen the existing RyR2 dysfunction and Ca^2+^ leak, not only affecting cardiac function, but also enhancing arrhythmogenesis. Accordingly, we present our viewpoint here that in the presence of RyR2 dysfunction and Ca^2+^ leak, further enhancing β-AR signaling is detrimental rather than beneficial, and β-AR desensitization/down-regulation in HF may be a self-preserving, adaptive process rather than a conventionally considered detrimental process. This viewpoint will be detailed in the following discussion.

### β-AR Desensitization/Down-Regulation and RyR2 Dysfunction/Ca^2+^ Leak Are Two Distinct Yet Related Abnormalities in HF

**Figure 1:**
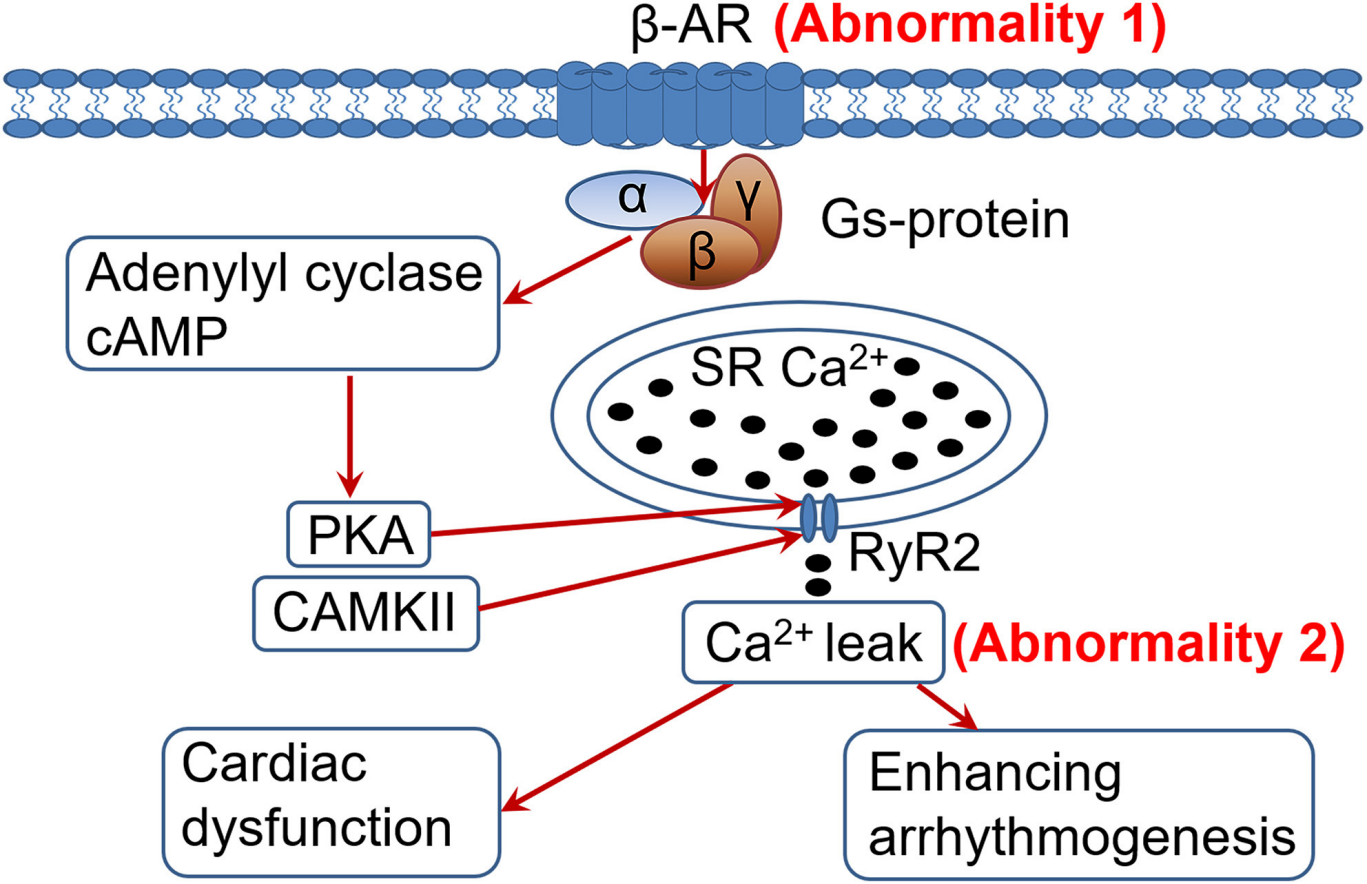
Heightened adrenergic signaling in the presence of cardiac ryanodine receptor (RyR2) dysfunction and Ca^2+^ leak is detrimental. In heart failure (HF), sustained adrenergic activation leads to hyperphosphorylation (among other posttranslational modifications) of the RyR2 by PKA and CaMKII (through PKA-independent signaling mediated by β1-AR activation), resulting in RyR2 dysfunction and pathological Ca^2+^ leak. Desensitization/down-regulationof the β-AR (abnormality 1) would diminish adrenergic signaling, alleviating RyR2 dysfunction and Ca^2+^ leak (abnormality 2) than they would otherwise. In contrast, enhancing adrenergic signaling in HF would worsen the RyR2 dysfunction and Ca^2+^ leak (abnormality 2), exacerbating cardiac dysfunction and arrhythmogenesis. PKA, protein kinase A; CaMKII, Ca^2+^/calmodulin-dependent protein kinase II; SR, sarcoplasmic reticulum; RyR2, cardiac ryanodine receptor.

In HF cardiomyocytes develop 2 distinct yet related abnormalities: β-AR desensitization/down-regulation and RyR2 dysfunction/Ca^2+^ leak (Figure 1). Both abnormalities are allegedly caused by the sustained sympathetic activation and β-AR signaling in HF (6, 14). β-AR desensitization/down-regulation (Figure 1, abnormality 1) in HF is well-characterized, which diminishes sympathetic signaling, cardiac functional capacity and contractile reserve, and is conventionally considered to be detrimental in HF progression (3, 11, 12). On the other hand, when RyR2 dysfunction and Ca^2+^ leak occur (abnormality 2), further enhancing β-AR signaling would worsen the existing RyR2 dysfunction and Ca^2+^ leak, leading to exacerbation of cardiac dysfunction and arrhythmogenesis (Figure 1). Accordingly, we postulate that β-AR desensitization/down-regulation, acting like an intrinsic β-AR blocker and by diminishing adrenergic signaling, can limit RyR2 dysfunction and Ca^2+^ leak from further deteriorating under sustained sympathetic adrenergic drive with persistent high-level of catecholamines in HF. In other words, β-AR desensitization/down-regulation in HF may be a self-preserving, adaptive process (a friend) rather than a conventionally considered detrimental/pathological process (a foe). This viewpoint is supported by the following several lines of evidence.

### Normal Ca^2+^ Signaling Is Essential in Mediating Physiological Sympathetic Response

Cardiac excitation-contraction coupling (ECC) refers to the conversion from electrical excitation (in the form of an action potential) into mechanical contraction. Briefly, cardiac action potential propagates along the cell membrane, including the specialized invaginations (T-tubules) (20). Depolarization activates voltage-gated L-type Ca^2+^ channels (LTCCs) on the T-tubules and allows Ca^2+^ to enter the cell. The entering Ca^2+^ then binds to and activates Ca^2+^ release channels (RyR2) on SR, releasing Ca^2+^ to cytoplasm from the SR (where Ca^2+^ is stored at high concentrations). This process by which Ca^2+^ entering cardiomyocyte through LTCCs activates RyR2 is known as Ca^2+^ induced Ca^2+^ release (21). The spike of Ca^2+^ concentration in cytoplasm initiates actin–myosin cross-bridging, causing muscle contraction (systole). Cardiac relaxation (diastole) is initiated when RyR2 close (terminating SR Ca^2+^ release) and Ca^2+^ are pumped back into the SR by the SERCA or extruded from the cell by the Na^+^/Ca^2+^ exchanger (NCX). This coordinated Ca^2+^ release and reuptake in cardiomyocytes are essential for ECC.

Normal Ca^2+^ signaling is required in mediating physiological sympathetic response. Sympathetic activation, mediated by the β-ARs-Gs-adenylyl cyclase-cAMP-protein kinase A (PKA) signaling pathway, activates and phosphorylates LTCCs, increasing inward Ca^2+^ current (22). The same cascade also phosphorylates RyR2 (23), increasing Ca^2+^ release from SR, which in turn increases cardiac contractility (14). Adrenergic signaling also activates SERCA, speeding up Ca^2+^ removal from cytosol, thus improving cardiac relaxation. This normal Ca^2+^ signaling is essential in realizing sympathetic adrenergic effects on the heart.

### Sustained Sympathetic Adrenergic Activation Leads to RyR2 Dysfunction and Ca^2+^ Leak, Enhancing Arrhythmogenesis in HF

It is known that ventricular arrhythmias are common in HF patients and about 50% of HF patients die due to sudden cardiac death (24). Accumulating evidence indicates that a pathogenic process of defective cardiac Ca^2+^ signaling plays a critical role in HF (16, 25, 26). As discussed, RyR2 open and close in a concerted manner during myocyte contraction and relaxation (27). In HF, chronic sympathetic adrenergic activation leads to unstable channels that leak Ca^2+^ during diastole because the channel cannot close properly (28). The RyR2 dysfunction in HF is reportedly caused by enhanced adrenergic signaling resulting in hyperphosphorylation, oxidation or S-nitrosylation of RyR2, etc., which results in Ca^2+^ leak by destabilizing the closed state of the channel (14). RyR2 dysfunction and Ca^2+^ leak can be observed in isolated cardiomyocytes as short, unsynchronized SR Ca^2+^ release events during diastole (29).

RyR2 dysfunction and Ca^2+^ leak could impair cardiac function and enhance arrhythmogenesis. Chronic Ca^2+^ leak via dysfunctional RyR2 depletes Ca^2+^ store in the SR, lowers Ca^2+^ transient amplitude in systole and reduces cardiac contractility (30). Ca^2+^ leak can lead to elevated Ca^2+^ levels in diastole, impairing myocyte relaxation. Thus, RyR2 dysfunction and Ca^2+^ leak could lead to both systolic and diastolic dysfunction. Moreover, Ca^2+^ leak and elevated diastolic Ca^2+^ levels increase Ca^2+^ extrusion via NCX, promoting an inward depolarizing current which is electrogenic and promotes triggered activity and arrhythmia formation (15). The detrimental effect of β-AR activation in HF is supported by evidence that treatment with β-AR agonists is associated with increased mortality in HF (31, 32), despite their short-term hemodynamic benefits. We have demonstrated recently that failing hearts are more vulnerable to develop both atrial and ventricular arrhythmias under sympathetic stimulation, despite less positive inotropy in HF (33, 34). Finally, this is also supported by the fact that β-AR blocker treatment can reduce cardiac arrhythmias and sudden cardiac death in HF (35).

### Sustained Sympathetic Activation Leads to β-ARs Desensitization/Down-Regulation in HF

HF is characterized by sympathetic activation and elevated blood catecholamine levels (3). As discussed, sympathetic adrenergic activation, mediated through β-AR signaling, increases cardiac contraction and relaxation. These responses are initially beneficial in supporting the failing heart by increasing cardiac output and sustaining circulation. However, long-term overstimulation of β-ARs eventually leads to loss of responsiveness to sympathetic signaling (β-ARs desensitization) and reduced density (number) of β-ARs on cell membrane (down-regulation), resulting in diminished β-AR signaling, and loss of contractile reserve. It has been reported that β-AR desensitization/down-regulation correlates with HF severity (11). These effects underlie the pathogenesis of HF (6).

β-AR desensitization in HF is caused by phosphorylation of β-ARs themselves by PKA and G-protein coupled receptor kinases (GRKs) (6). GRK activity has been found to be augmented by prolonged β-AR activation under catecholamine exposure. GRK levels are elevated in the setting of HF and contribute to its pathogenesis (36). In addition, GRK activation and β-AR uncoupling can lead to β-ARs (mainly β1-ARs) internalization (β1-ARs shifted from the sarcolemma membrane into cytosolic compartments), contributing to β-ARs down-regulation (6). It has been reported that prolonged stimulation of β-AR signaling can induce β-AR down-regulation even without HF (37).

### In the Presence of Abnormal Ca^2+^ Handling, Further Enhancing Adrenergic Signaling Could Be Detrimental Rather Than Beneficial in HF

RyR2 dysfunction and Ca^2+^ leak in HF are caused by sustained adrenergic signaling (14). It would be obvious that further activation of the adrenergic signaling can further worsen RyR2 dysfunction and Ca^2+^ leak (Figure 1). In HF the Ca^2+^ leak is usually less at rest. With enhanced adrenergic signaling during sympathetic stress, the RyR2 dysfunction and Ca^2+^ leak would deteriorate (14). This would exacerbate cardiac dysfunction and promote arrhythmogenesis (15). As shown in the figure, the stronger the β-AR signaling, the worse the RyR2 dysfunction and Ca^2+^ leak, and thus more arrhythmogenesis. It could be speculated that if there were no β-AR desensitization/down-regulation in HF, there would be an even greater risk of developing arrhythmias and sudden cardiac deaths in HF patients due to persistent sympathetic activation and elevated catecholamine levels. Our recent findings that failing hearts are more vulnerable to develop arrhythmias under sympathetic stimulation (33, 34) support this concept. In line with this, β-AR agonist treatment is associated with increased mortality in HF (31, 32) and β-AR blocker treatment can reduce cardiac arrhythmias and sudden cardiac death in HF (35).

Thus, in the presence of RyR2 dysfunction and Ca^2+^ leak in HF, β-ARs desensitization/down-regulation, by diminishing adrenergic signaling, helps to attenuate RyR2 dysfunction and Ca^2+^leak, protecting the heart from developing serious cardiac arrhythmias and sudden cardiac death. In other words, β-ARs desensitization/down-regulation in HF may act in a way similar to a β-AR blocker, attenuating adrenergic signaling, which is beneficial especially under heightened sympathetic activation with persistent high-level of catecholamines seen in HF. From this point of view, β-AR desensitization/down-regulation in HF is a self-preserving, adaptive process (a friend) rather than a detrimental, pathological process (a foe), as conventionally considered.

## Conclusion

β-AR desensitization/down-regulation is one of the many characteristic abnormalities seen in HF that is conventionally considered to be detrimental in HF progression (3, 11, 12). However, emerging evidence indicates that this concept may be too simplistic and may not be correct. Despite the fact that β-AR desensitization/down-regulation in HF diminishes adrenergic signaling and cardiac contractile reserve, this process may act as an intrinsic β-AR blocker, protecting the heart from developing more severe ventricular arrhythmias and sudden cardiac death. In line with this, new medications designed to re-sensitize/up-regulate β-ARs have to also normalize Ca^2+^ handling to be beneficial. Otherwise, simply enhancing adrenergic signaling without correcting RyR2 dysfunction (like β-AR agonists) may exacerbate RyR2 dysfunction and Ca^2+^ leak, increasing the arrhythmia risk and sudden cardiac death in HF. It should be noted that β-AR blocker treatment in HF is associated with normalization of RyR2 function and Ca^2+^ signaling (38), besides re-sensitizing/up-regulating the β-ARs.

## Data Availability Statement

The original contributions presented in the study are included in the article/supplementary material, further inquiries can be directed to the corresponding author/s.

## Author Contributions

YZ contributed to conception of the manuscript. AM and KA wrote the first draft of the manuscript. All authors contributed to manuscript revision, read, and approved the submitted version.

## Conflict of Interest

The authors declare that the research was conducted in the absence of any commercial or financial relationships that could be construed as a potential conflict of interest.

## Publisher's Note

All claims expressed in this article are solely those of the authors and do not necessarily represent those of their affiliated organizations, or those of the publisher, the editors and the reviewers. Any product that may be evaluated in this article, or claim that may be made by its manufacturer, is not guaranteed or endorsed by the publisher.
